# Hypoxia-inducible factor 1-alpha inhibition mitigates inflammatory responses induced by H9N2 avian influenza virus

**DOI:** 10.1128/spectrum.02680-25

**Published:** 2026-08-25

**Authors:** Suying Zhu, Beibei Li, Yaqi Wang, Yuhan Tian, Jiayue Zhang, Hongshan Wang

**Affiliations:** 1 College of Agriculture and Bioengineering, Heze University, Heze, China; University of São Paulo, São Paulo, Brazil

**Keywords:** H9N2 avian influenza virus, hypoxia-inducible factor 1-alpha, Sotetsuflavone, inflammation

## Abstract

H9N2 avian influenza virus (AIV) poses significant threats to poultry and public health, with the potential to contribute to the emergence of more virulent influenza strains. Hypoxia-inducible factor 1-alpha (HIF-1α) plays a crucial role in viral pathogenesis, immune regulation, and inflammation, making it a promising target for therapeutic intervention. Here, we explored the involvement of HIF-1α in H9N2 AIV pathogenesis and associated inflammatory responses. Our data revealed that HIF-1α exacerbates inflammation induced by H9N2 AIV infection through its regulation of mitochondrial fitness. Additionally, we investigated Sotetsuflavone (Seo), a naturally derived flavonoid, for its potential to modulate inflammatory responses following H9N2 infection. Molecular docking coupled with surface plasmon resonance assays indicated that Seo exhibits robust binding affinity to HIF-1α, implying a direct inhibitory mechanism. Subsequent *in vitro* studies demonstrated that Seo greatly reduced the production of inflammatory cytokines, including IL-1β, IL-6, and TNF-α, through suppressing the HIF-1α signaling pathway in HD11 cells infected with H9N2 AIV. Correspondingly, *in vivo* experiments established that Seo administration significantly ameliorated pulmonary inflammation in H9N2-infected mice, while also enhancing alveolar epithelial repair, as evidenced by increased expression of type II alveolar epithelial cell markers Abca3 and Sftpb. Collectively, these findings identify Seo as a potent inhibitor of HIF-1α, highlighting its therapeutic potential in mitigating inflammation and lung damage associated with influenza virus infection.

**IMPORTANCE:**

H9N2 avian influenza virus triggers inflammatory lung injury via hypoxia-inducible factor-1 alpha (HIF-1α)-mediated dysregulation of mitochondrial function. The natural flavonoid Sotetsuflavone (Seo) directly binds and inhibits HIF-1α, as confirmed by molecular docking and surface plasmon resonance assays. In H9N2-infected HD11 cells, Seo suppresses HIF-1α signaling, reducing IL-1β, IL-6, and TNF-α production. *In vivo*, Seo treatment attenuates pulmonary inflammation and enhances alveolar epithelial repair in mice, upregulating type II pneumocyte markers (Abca3 and Sftpb). These findings identify Seo as a promising HIF-1α-targeted therapeutic for influenza-associated immunopathology.

## INTRODUCTION

The H9N2 subtype of avian influenza virus (AIV) is recognized as a notable pathogen, significantly impacting animal and public health (1, 2). Recently, H9N2 AIV has emerged as the predominant avian influenza strain circulating among poultry worldwide, resulting in considerable economic repercussions within the poultry sector (3). Infection in chickens commonly manifests as severe loss of appetite, heightened vulnerability to secondary bacterial infections, and marked decreases in egg production (4, 5). Thus, effective antiviral therapeutics and vaccines against H9N2 AIV are urgently required.

Hypoxia-inducible factor-1 alpha (HIF-1α), a critical transcriptional regulator under hypoxic conditions, modulates numerous cellular processes (6). Beyond its traditional role in managing oxygen balance, growing evidence underscores its involvement in viral pathogenesis through regulation of immune responses, viral replication, and inflammatory signaling (7, 8). For instance, HIF-1α facilitates reactivation of Marek’s disease virus (9), and influenza A virus-induced glycolytic activity enhances viral propagation via reactive oxygen species (ROS)-mediated activation of the HIF-1α pathway (10). Additionally, HIF-1α promotes innate immune activation through upregulation of inflammatory cytokines like IL-1β and TNF-α (8). Given its pivotal role in viral infections and inflammation, HIF-1α represents an attractive therapeutic target, particularly in cases of excessive virus-induced inflammatory responses. Nonetheless, the specific involvement of HIF-1α in H9N2 AIV infection has not been previously clarified.

Several compounds have been identified that effectively inhibit H9N2 AIV. The antiviral peptide P1, for example, significantly reduces viral replication *in vitro* and *in vivo* through interactions with the hemagglutinin glycoprotein, thereby preventing viral entry into host cells (11). Similarly, *furanocoumarins* extracted from *Angelica dahurica* effectively disrupt early viral replication stages (12), while *Belamcanda* extract notably diminishes inflammatory responses triggered by H9N2 AIV (13). Sotetsuflavone (Seo), a natural flavonoid, has garnered attention due to its anti-inflammatory and antiviral capabilities. Seo ameliorates colitis resembling Crohn’s disease by mitigating intestinal damage caused by M1 macrophages via inhibition of the c-Jun N-terminal kinase and mitogen-activated protein kinase pathways (14). Additionally, Seo efficiently suppresses dengue virus replication by inhibiting NS5 RNA-dependent RNA polymerase (15). However, its potential role in alleviating pathology induced by H9N2 AIV has not been previously explored.

Here, our results showed that HIF-1α mediates the inflammation induced by H9N2 AIV in chicken macrophages. Additionally, HIF-1α is critical for maintaining mitochondrial homeostasis by enhancing respiratory capacity during H9N2 AIV infection. Notably, Seo functions as an effective HIF-1α inhibitor and significantly reduces viral inflammation both *in vitro* and *in vivo*. Therefore, Seo is a potential therapeutic drug for controlling H9N2 AIV infection.

## MATERIALS AND METHODS

### Animal infection

Female wild-type C57BL/6 mice, aged 6–8 weeks or 16 months, were obtained from Senwell Experimental Animals Co., Ltd. (Chengdu, China) and maintained under controlled conditions (25 ± 1°C, relative humidity of 55 ± 5%, and a 12-h light/12-h dark cycle). To evaluate the *in vivo* inhibitory effects of Sotetsuflavone (Seo), mice received intraperitoneal injections of either PBS or Seo (0.1 mg per mouse) for three consecutive days. On the third day, mice were anesthetized and intranasally inoculated with 50 μL of influenza virus at a dose of 10 × LD₅₀. After viral exposure, animals were observed on a daily basis to evaluate clinical symptoms and disease progression.

### Animal histopathology

Mice were sacrificed at 10 days postinfection, and the left lobe of the lung was fixed with 4% paraformaldehyde (PFA). Histological analysis was performed on formalin-fixed, paraffin-embedded tissue sections (5 μm) stained with hematoxylin and eosin (H&E). Slides were examined under a light microscope. Inflammatory lesions were evaluated based on the presence, distribution, and severity of immune cell infiltration, edema, epithelial damage, and tissue architecture disruption.

### Cell culture and virus propagation

The HD11 avian macrophage cell line was cultured in RPMI 1640 medium (Sigma-Aldrich, USA) supplemented with 10% heat-inactivated fetal bovine serum (Gibco, USA), 100 IU/mL penicillin, and 100 μg/mL streptomycin under standard culture conditions of 37°C and 5% CO_2_ in a humidified atmosphere. The H9N2 influenza A virus strain maintained in the laboratory repository was propagated in 9-day-old specific pathogen-free (SPF) embryonated chicken eggs. Following a 72-h incubation period at 37°C, the viral stocks were harvested and stored at −80°C.

### Virus titration

Viral culture supernatant containing virus was serially diluted tenfold and introduced into 9-day-old specific pathogen-free (SPF) embryonated eggs. After incubation for 60 h, the allantoic fluid was harvested and analyzed for hemagglutination activity using chicken red blood cells to evaluate viral replication efficiency. Viral titers, expressed as log₁₀ 50% egg infectious doses (EID_50_) per milliliter, were determined through triplicate experiments and calculated using the Reed–Muench method (16).

### Plasmid construction

The mouse HIF-1α gene was amplified by reverse transcription polymerase chain reaction (RT-PCR) using total RNA isolated from HD11 cells. Forward primer: 5′-CGGAATTCATGTCGACAGCCTAACAGTTG-3′; Reverse primer: 5′-CCGCTCGAGTCAAGTCTAGCAGTTCTTGTG-3′. The HIF-1α gene was cloned into the pCMV-Flag vector (restriction enzyme site: EcoRI and XhoI). Recombinant plasmids were verified by sequencing.

### Cell transfection

A total of 1 × 10⁵ cells were seeded into 12-well culture plates, reaching ~80% confluency before the experimental procedures. After a 24-h incubation, cell transfection was performed using 3 μg of specific plasmid constructs per well with Lipofectamine 2000 reagent (Invitrogen, USA), following the manufacturer's protocol. At 24 h after transfection, cells were infected with virus at a multiplicity of infection (MOI) of 1. Samples were then collected at indicated time points for downstream analysis.

### RNA interference

Cells were seeded into 12-well culture plates at an initial density of 50%. On the following day, siRNA transfection was conducted using Lipofectamine RNAiMAX (Invitrogen, USA) in accordance with the manufacturer’s guidelines. All siRNAs used in this study were synthesized from GenePharma (Shanghai, China). After a 36-h incubation, cells were infected with H9N2 IAV at an MOI of 1. Samples were collected at indicated time points for subsequent analyses. The sequences of siRNA targeting HIF-1α are 5′-CUAACUGGA CACAGUGUGU-3′ and 5′-ACACACUGUGUCCAGUUAG-3′.

### Western blotting

Cells were lysed in a 1:1 mixture of RIPA buffer and 1 × SDS loading buffer, then denatured by boiling at 100°C for 5 min. Samples were then subjected to SDS-PAGE and transferred onto NC membranes. Membranes were blocked with 5% nonfat dry milk for 1 h at room temperature, followed by three washes with TBST (5 min per wash) and incubated with specific primary antibodies: rabbit anti-HIF-1α (36169, CST, USA) and mouse anti-actin (66009-1-Ig, Proteintech, China). Following another round of washes, immunoreactive bands were detected using the ECL kit (Beyotime, China) and analyzed with Image J software (NIH, USA)

### Enzyme-linked immunosorbent assay

The levels of IL1β, IL-6, and TNF-α in the supernatant were quantified using commercially available ELISA kits, including the Mouse IL-1β ELISA Kit (PI301), Mouse IL-6 ELISA Kit (PI326), and Mouse TNF-α ELISA Kit (PT512) from Beyotime (China), according to the manufacturer’s instructions.

### Quantitative reverse transcription polymerase chain reaction

Total RNA was isolated from samples using Trizol reagent (TaKaRa, Japan). The concentration and purity of RNA were assessed using NanoDrop (Thermo Scientific, USA). Complementary DNA (cDNA) was then synthesized using PrimeScript RT Master Mix (TaKaRa, Japan). Quantitative real-time PCR (qPCR) was performed using TB Green Premix Ex Taq II (TaKaRa, Japan). The conditions were as follows: 95°C for 10 min, followed by 40 cycles of denaturation (95°C, 15 s), annealing (specific temperature, 30 s), and extension (72°C, 30 s). Relative mRNA expression levels were determined using the 2^−ΔΔCt^ method (17). The primers used are listed in Table 1.

**TABLE 1:** Primer sequences of target genes

Gene	Primer sequences (5′−3′)
IL-1β	F: GGGCATCAAGGGCTACAA R: CTGTCCAGGCGGTAGAAGA
IL-6	F: AAATCCCTCCTCGCCAATCT R: CCCTCACGGTCTTCTCCATAAA
IL-10	F: GCTGAGGGTGAAGTTTGAG R: CAGGTGAAGAAGCGGTGA
TNF-α	F: CCTACCCTGTCCCACAACCT R: TGAACTGGGCGGTCATAGAA
β-actin	F: ATGGCTCCGGTATGTGCAAG R: CAACCATCACACCCTGATGTC

### Oxygen consumption rate assessment

The oxygen consumption rate (OCR) was assessed in real time using a Seahorse XFp Analyzer (Agilent, USA). After viral infection or transfection, HD11 cells (1 × 10⁵ cells per well) were seeded in Seahorse XFp Cell Culture Miniplates and incubated overnight at 37°C in a 5% CO_₂_ atmosphere. The next day, cells were washed twice and then preincubated at 37°C for 1 h in an unbuffered assay medium (pH 7.4, Agilent Technologies) under CO_₂_-free conditions. OCR measurements were taken under basal conditions and after sequential injections of metabolic inhibitors: 1 mM oligomycin (HY-N6782, MCE, USA), 1.5 mM carbonyl cyanide 4 (trifluoromethoxy)phenylhydrazone (FCCP) (HY-100410, MCE, USA), and a combination of 0.5 mM rotenone (HY-B1756, MCE, USA) with 0.5 mM antimycin A (A8674, Sigma-Aldrich, USA). Data analysis was conducted using Wave Desktop software (Agilent, USA).

### Network pharmacology analysis

The chemical structure of Seo was imported into the STITCH database (https://cn.string-db.org/) to predict overlapping target genes associated with H9N2 IAV and H5N1. To evaluate the importance of shared targets, they were input into the STRING database (https://string-db.org/) for network topology, using a combined score cutoff of >0.95. Network topology parameters, including betweenness centrality (BC), closeness centrality (CC), degree centrality (DC), eigenvector centrality (EC), network centrality (NC), and local average connectivity (LAC), were calculated to assess node significance within the network. Higher parameter values indicated greater node importance. Subsequently, a protein-protein interaction (PPI) network was constructed. Node-degree distribution diagrams were generated using the NetworkAnalyzer plugin in Cytoscape (version 3.10.1), treating the network as an undirected graph (18). Finally, functional enrichment analyses, including Kyoto Encyclopedia of Genes and Genomes (KEGG) pathway analysis and Gene Ontology (GO) annotation, were performed, with statistical significance defined as *P* < 0.05.

### Molecular docking

Molecular docking was performed to analyze the interaction between Seo and mouse HIF-1α. The structure of Sotetsuflavone was optimized and saved in PDB format. The crystal structure of mouse HIF-1α was retrieved from the Protein Data Bank and prepared. Docking simulations were conducted using AutoDock Vina, with a grid box centered on the HIF-1α active site. Docking results were analyzed using PyMOL to identify binding affinity and key interactions between Sotetsuflavone and HIF-1α residues (19).

### Surface plasmon resonance

Surface plasmon resonance (SPR) binding assays were performed on a Biacore T200 instrument (GE Healthcare) at 25°C. Phosphate-buffered saline containing 0.05% Tween 20 (PBST) was used as the running buffer and filtered through a 0.22 μm membrane prior to use. The recombinant protein was immobilized onto a CM5 sensor chip via standard amine coupling chemistry. Briefly, the chip surface was activated with a 1:1 mixture of 0.4 M N-ethyl-N′-(3-dimethylaminopropyl) carbodiimide and 0.1 M N-hydroxysuccinimide for 420 s at 10 μL/min, followed by injection of the protein solution (30 μg/mL in 10 mM sodium acetate, pH 4.5) at 10 μL/min until the desired immobilization level was achieved. Unreacted sites were blocked with 1 M ethanolamine (pH 8.5) for 420 s at 10 μL/min. Seo was serially diluted in running buffer containing 0.1% DMSO, and each concentration was injected over the sensor surface at 30 μL/min for a 60 s association phase, followed by a 60 s dissociation phase. The surface was regenerated, if necessary, using PBST washes. Sensorgrams were recorded in real time, and kinetic parameters (association rate constant, dissociation rate constant, and equilibrium dissociation constant) were determined using Biacore 1K evaluation software with a 1:1 Langmuir binding model. All experiments were performed in triplicate to ensure reproducibility.

### Statistical analysis

Data are presented as the mean ± SD. The Student’s *t*-test was used for all statistical analyses with GraphPad Prism 10 software (CA, USA). Differences between groups were considered significant when the *P* < 0.05 (*).

## RESULTS

### HIF-1α deficiency suppresses the inflammatory response induced by H9N2 AIV in HD11 cells

**Fig 1:**
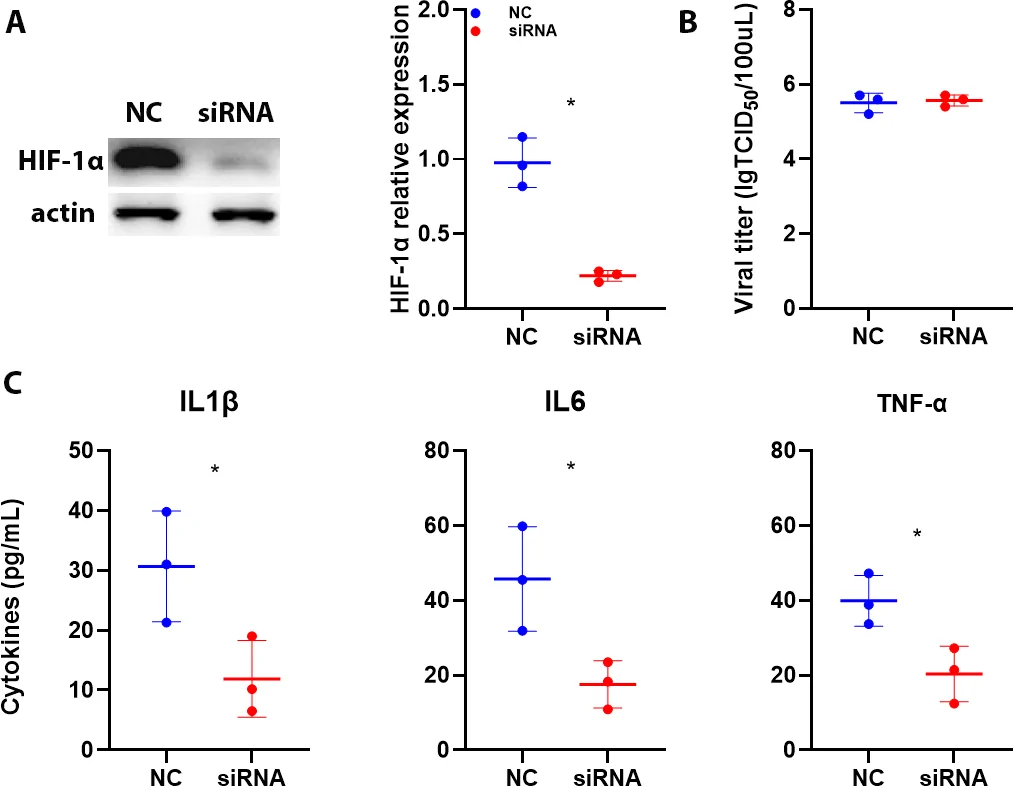
HIF-1α deficiency mitigates the inflammation response induced by H9N2 infection. HD11 cells were transfected with negative control siRNA (NC) or siRNA targeting HIF-1α (siRNA). After 36 h, cells were infected with H9N2. (**A**) The depletion of HIF-1α was confirmed by Western blotting. (**B**) Viral titers were determined. (**C**) The levels of inflammatory cytokines IL-1β, IL-6, and TNF-α were measured using ELISA. Data are presented as means ± SEM. *P < 0.05.

To elucidate the role of HIF-1α in the pathogenesis of H9N2 AIV, RNA interference was employed to reduce HIF-1α expression in HD11 cells, followed by infection with H9N2 AIV. As shown in Fig. 1A, the HIF-1α-deficient HD11 cell line was successfully established. Our results further showed that viral titers were comparable between the control and HIF-1α-deficient groups, suggesting that HIF-1α is not directly involved in viral replication (Fig. 1B). Given that H9N2 AIV induces excessive inflammation in poultry, we evaluated the inflammatory response. Our results revealed that HIF-1α depletion significantly decreased the expression levels of key pro-inflammatory cytokines, including interleukin 1β (IL1β), interleukin 6 (IL 6), and tumor necrosis factor-α (TNF-α; *P* < 0.05; Fig. 1C). These findings indicate that HIF-1α suppression attenuates inflammation during H9N2 AIV infection.

### Overexpressing HIF-1α promotes the inflammation during H9N2 AIV infection in HD11 cells

**Fig 2:**
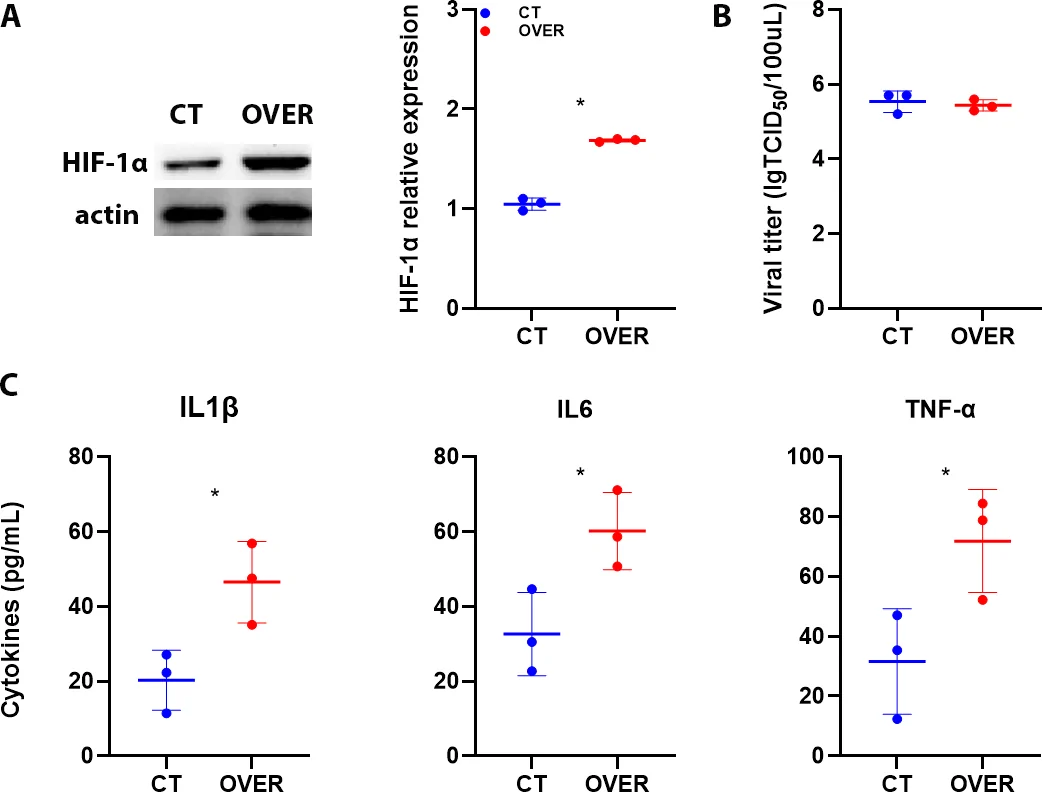
Ectopic expression of HIF-1α exacerbates viral-induced inflammation. HD11 cells were transfected with empty vector (CT) or a plasmid encoding HIF-1α (OVER). After 24 h, cells were infected with H9N2. (**A**) The expression level of HIF-1α was confirmed by WB. (**B**) Viral titers were determined. (**C**) The levels of inflammatory cytokines IL-1β, IL-6, and TNF-α were measured using ELISA. Data are presented as means ± SEM. *P < 0.05.

To further confirm the pro-inflammatory effect of HIF-1α upon H9N2 IAV infection, an HIF-1α overexpression plasmid was transfected into HD11 cells and verified via Western blot (WB; Fig. 2A). Consistently, replenishment of HIF-1α had a negligible effect on H9N2 IAV replication, as evidenced by comparable viral titers between control and HIF-1α-overexpressing cells (Fig. 2B). In contrast to HIF-1α deficiency, upregulated HIF-1α significantly enhanced the levels of inflammatory cytokines, including IL-1β, IL-6, and TNF-α (*P* < 0.05; Fig. 2C). Collectively, these findings demonstrate that HIF-1α positively regulates H9N2 IAV-induced inflammation in HD11 cells.

### HIF-1α deficiency contributes to mitochondria homeostasis during H9N2 AIV infection in HD11 cells

**Fig 3:**
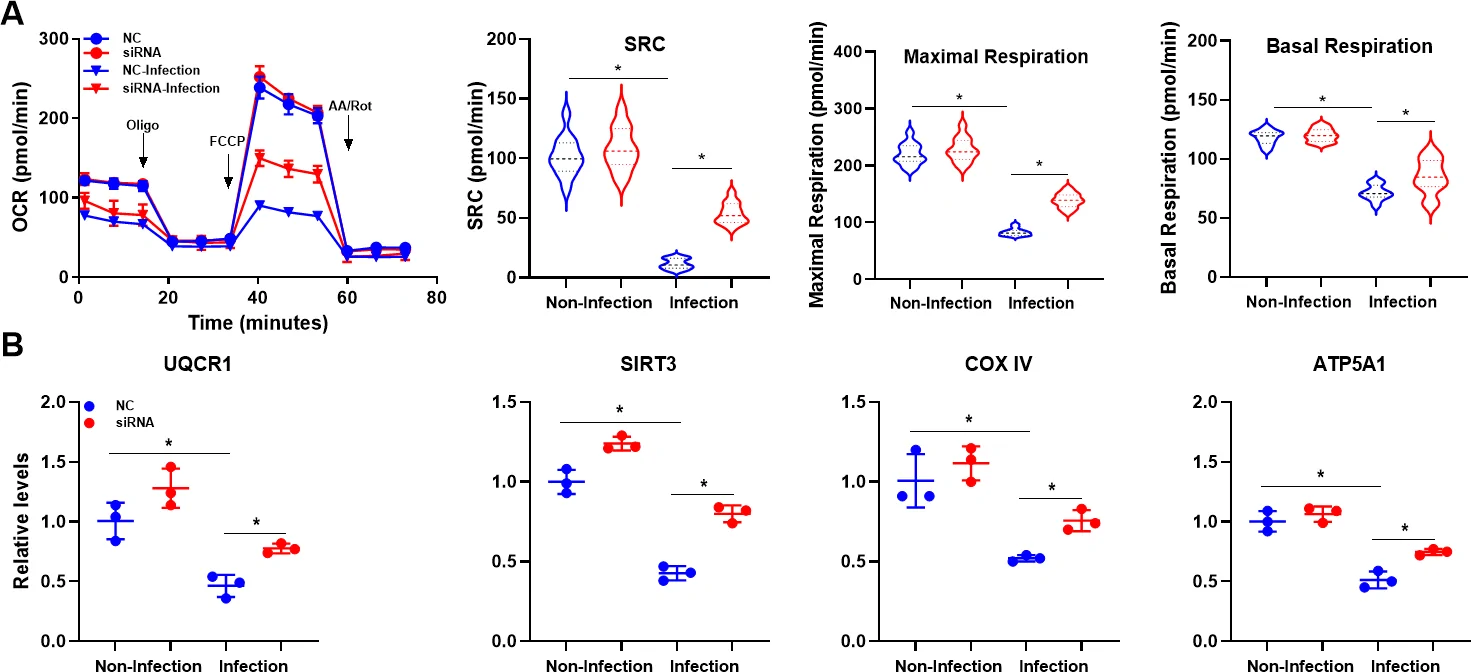
HIF-1α depletion maintains mitochondrial fitness during H9N2 infection. HD11 cells were transfected with negative control siRNA (NC) or siRNA targeting HIF-1α (siRNA). After 36 h, cells were infected with H9N2. (**A**) HD11 cells were treated with oligomycin, carbonyl cyanide-4-(trifluoromethoxy) phenylhydrazone (FCCP), and antimycin A/rotenone to measure the oxygen consumption rate (OCR). Spare respiratory capacity (SRC), maximal respiratory capacity, and basal respiration were quantified. (**B**) RT-qPCR was conducted to assess the expression levels of genes related to mitochondrial OXPHOS complexes. Data are presented as means ± SEM. *P < 0.05.

Extensive evidence indicates that mitochondrial dysfunction contributes to inflammation (20). To further investigate this, we conducted Seahorse analysis to measure the OCR of mitochondria. As shown in Fig. 3A, H9N2 IAV infection significantly reduced spare respiratory capacity (SRC), maximal respiratory capacity, and basal respiration in HD11 cells compared to the control group (*P* < 0.05). Additionally, we assessed mitochondrial homeostasis by examining key genes related to oxidative phosphorylation (OXPHOS) complexes, including UQCR1 (ubiquinol-cytochrome C reductase subunit I, Complex III), COX IV (Cytochrome C oxidase complex subunit IV), ATP5A1 (ATP synthase F1 subunit alpha), and SIRT3 (Sirtuin 3). Following H9N2 IAV infection, the mRNA levels of UQCR1, COX IV, ATP5A1, and SIRT3 were significantly downregulated (*P* < 0.05). Moreover, HIF-1α depletion abolished virus-induced inflammation (Fig. 3B). Therefore, our data indicate that HIF-1α depletion promotes mitochondrial health during H9N2 IAV infection.

### HIF-1α overexpression exacerbates mitochondria dysfunction induced by H9N2 AIV infection in HD11 cells

**Fig 4:**
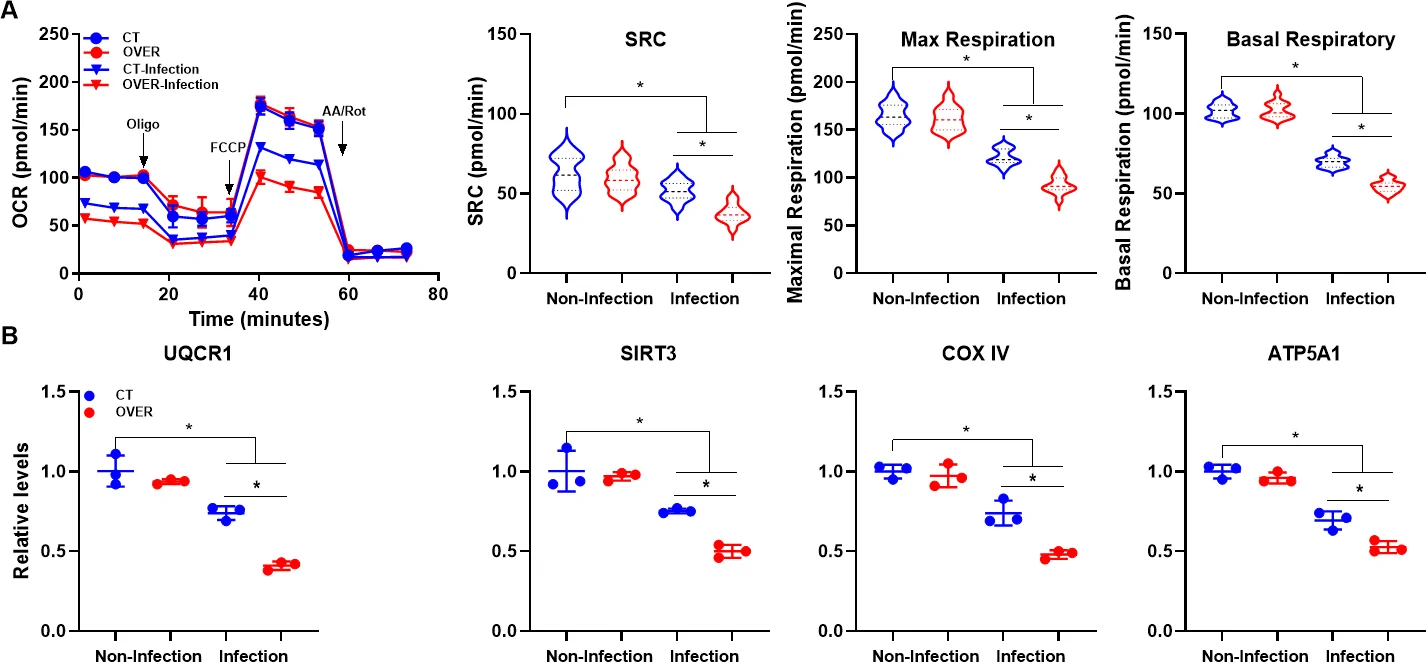
HIF-1α overexpression reduces mitochondrial fitness during H9N2 infection. HD11 cells were transfected with an empty vector (CT) or a plasmid encoding HIF-1α (OVER). After 24 h, cells were infected with H9N2. (**A**) HD11 cells were treated with oligomycin, carbonyl cyanide-4-(trifluoromethoxy) phenylhydrazone (FCCP), and antimycin A/rotenone to measure the OCR. SRC, maximal respiratory capacity, and basal respiration were quantified. (**B**) RT-qPCR was conducted to assess the expression levels of genes related to mitochondrial OXPHOS complexes. Data are presented as means ± SEM. *P < 0.05.

To further confirm the role of HIF-1α in regulating mitochondrial homeostasis, we performed HIF-1α overexpression experiment. Consistent with previous findings, H9N2 IAV infection significantly reduced SRC, maximal respiratory capacity, and basal respiration in HD11 cells compared to the control group (*P* < 0.05). However, HIF-1α overexpression further exacerbated these reductions following viral infection (Fig. 4A). Additionally, we assessed the expression of key OXPHOS complex marker genes. As shown in Fig. 4B, UQCR1, COX IV, ATP5A1, and SIRT3 expression levels were decreased after H9N2 IAV infection (*P* < 0.05). Notably, HIF-1α overexpression further downregulated the mRNA levels of these genes (*P* < 0.05). Overall, these findings suggest that HIF-1α plays a crucial role in mitochondrial homeostasis during H9N2 IAV infection.

### Screening of Sotetsuflavone as a potential small molecule targeting HIF-1α and inflammation

**Fig 5:**
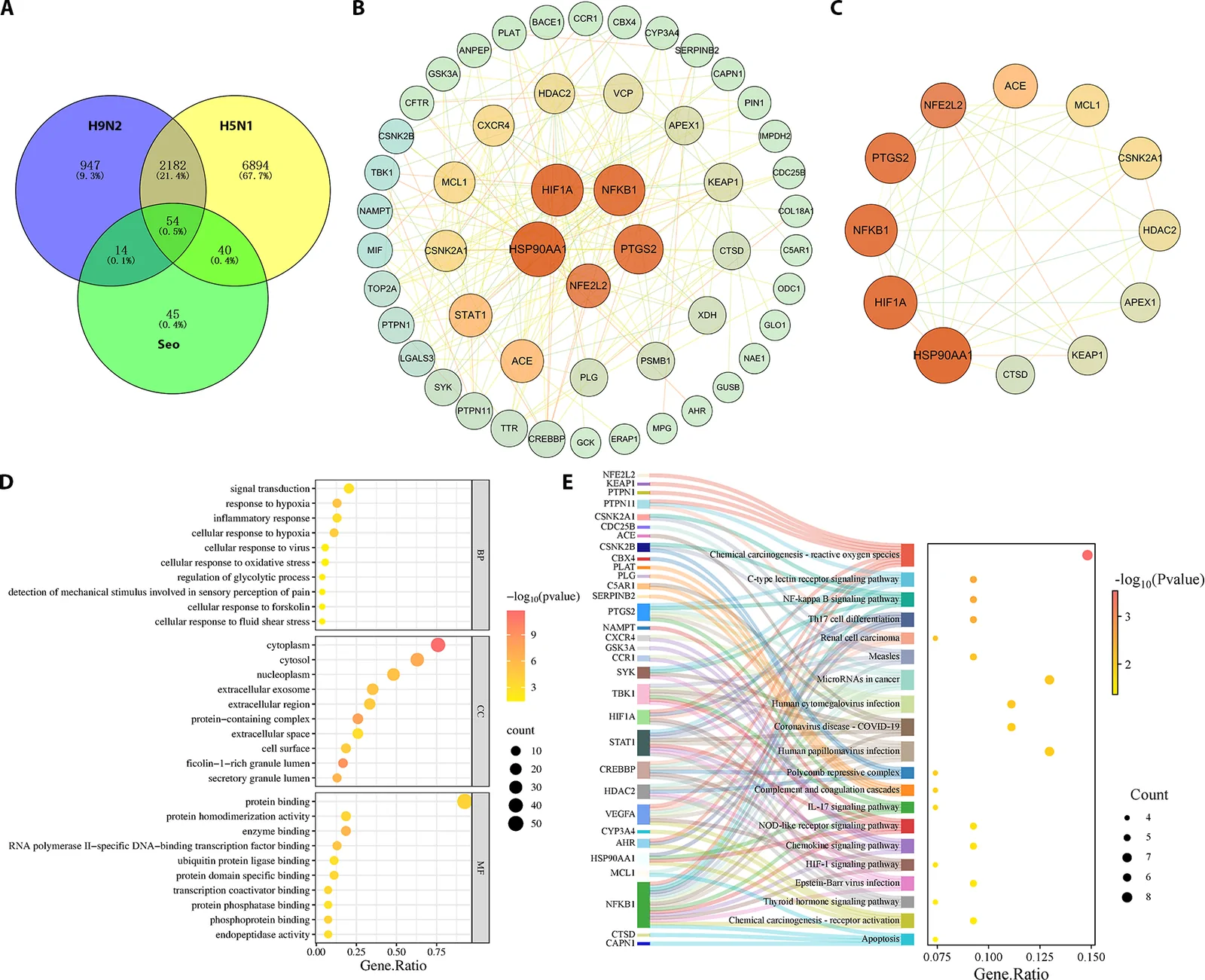
Network pharmacology analysis of Seo and H9N2, H5N1. (**A**) Overlap of Seo potential targets and H9N2 or H5N1-related targets. (**B and C**) The PPI network of the Seo-H9N2/H5N1 crossover genes. (**C and D**) Enrichment analysis and gene-pathway network analysis. (**D**) GO enrichment analysis of core targets of Seo in the treatment of H9N2/H5N1. (**E**) Bubble chart of KEGG pathway annotation.

Recent research indicates that Seo can downregulate HIF-1α expression (21). This raises the possibility that Seo may influence H9N2 AIV infection by targeting HIF-1α. To explore this hypothesis, we conducted a network pharmacology analysis, incorporating both H9N2 and H5N1 strains to examine potential broad-spectrum regulatory effects. As illustrated in Fig. 5A, 54 shared targets were identified. Additionally, PPI analysis ranked HIF-1α among the top five key genes (Fig. 5B and C). Further GO enrichment analysis, covering biological processes, cellular components, and molecular functions, revealed that the identified genes were primarily linked to hypoxia, inflammation, and cellular stress (Fig. 5D). Consistently, KEGG pathway analysis highlighted the enrichment of HIF-1α-related signaling pathways (Fig. 5E). These results suggest that Seo may modulate influenza virus infections by targeting HIF-1α and associated inflammatory responses.

### Sotetsuflavone serves as an effective HIF-1α inhibitor to mitigate H9N2-induced inflammation

**Fig 6:**
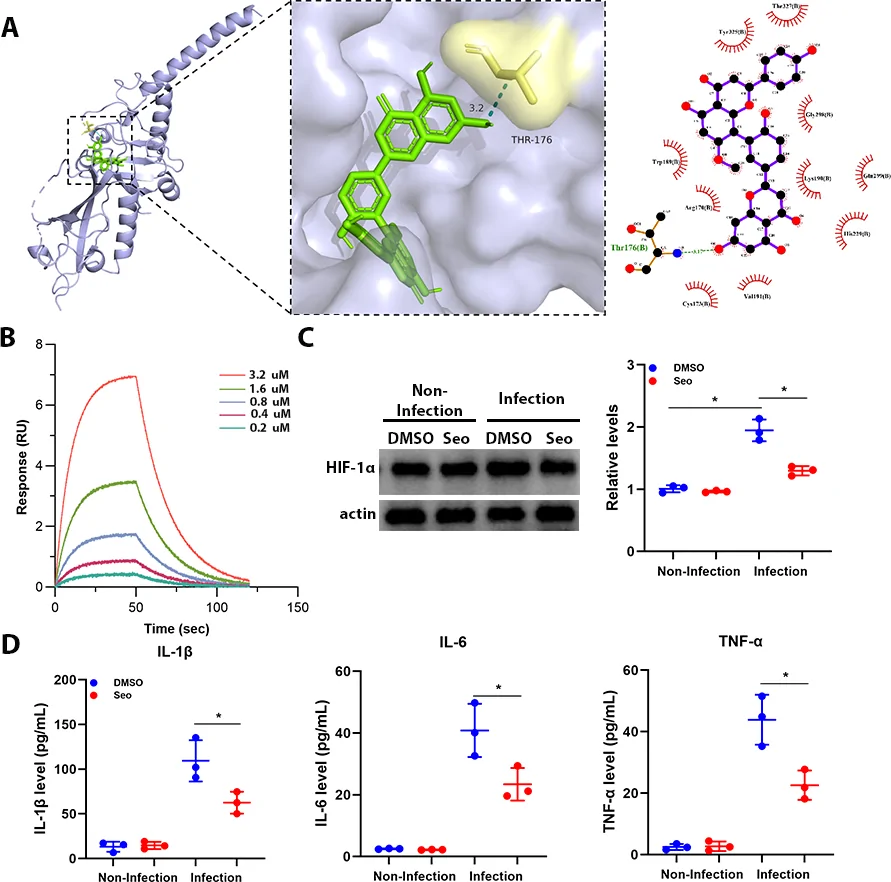
Seo functions as a potent HIF-1α inhibitor to mitigate H9N2-induced inflammation. (**A**) Molecular docking of Seo with HIF-1α. (**B**) The binding affinity between Seo and HIF-1α was detected by SPR. (**C**) HD11 cells were incubated with DMSO or AST IV overnight, followed by H9N2 infection. The HIF-1α protein level was detected by WB. (**D**) Gene expression levels of IL-1β, IL-6, and TNF-α in HD11 cells infected with or without H9N2 in the presence of DMSO or Seo overnight *in vitro*. Data are presented as means ± SEM. *P < 0.05.

To further assess the potential interaction between HIF-1α and Seo, molecular docking analysis was conducted. The results revealed a strong binding affinity, with a docking score of −9.3 (Fig. 6A). Additionally, SPR analysis estimated the equilibrium dissociation constant (K_D) at 5.1 × 10⁻⁷ M (Fig. 6B), suggesting specific binding and a concentration-dependent interaction. Furthermore, viral infection was found to elevate HIF-1α levels, whereas Seo treatment significantly suppressed its expression (Fig. 6C; *P* < 0.05). These findings confirm Seo as an effective HIF-1α inhibitor. To further examine its impact, HD11 cells were pretreated with Seo before H9N2 AIV infection. As illustrated in Fig. 6D, Seo markedly reduced the production of inflammatory cytokines, as evidenced by the decreased levels of IL-1β, IL-6, and TNF-α (*P* < 0.05). In summary, Seo effectively inhibits HIF-1α and mitigates H9N2-induced inflammation.

### Sotetsuflavone alleviates lung inflammation while promoting lung repair in mice during H9N2 infection

**Fig 7:**
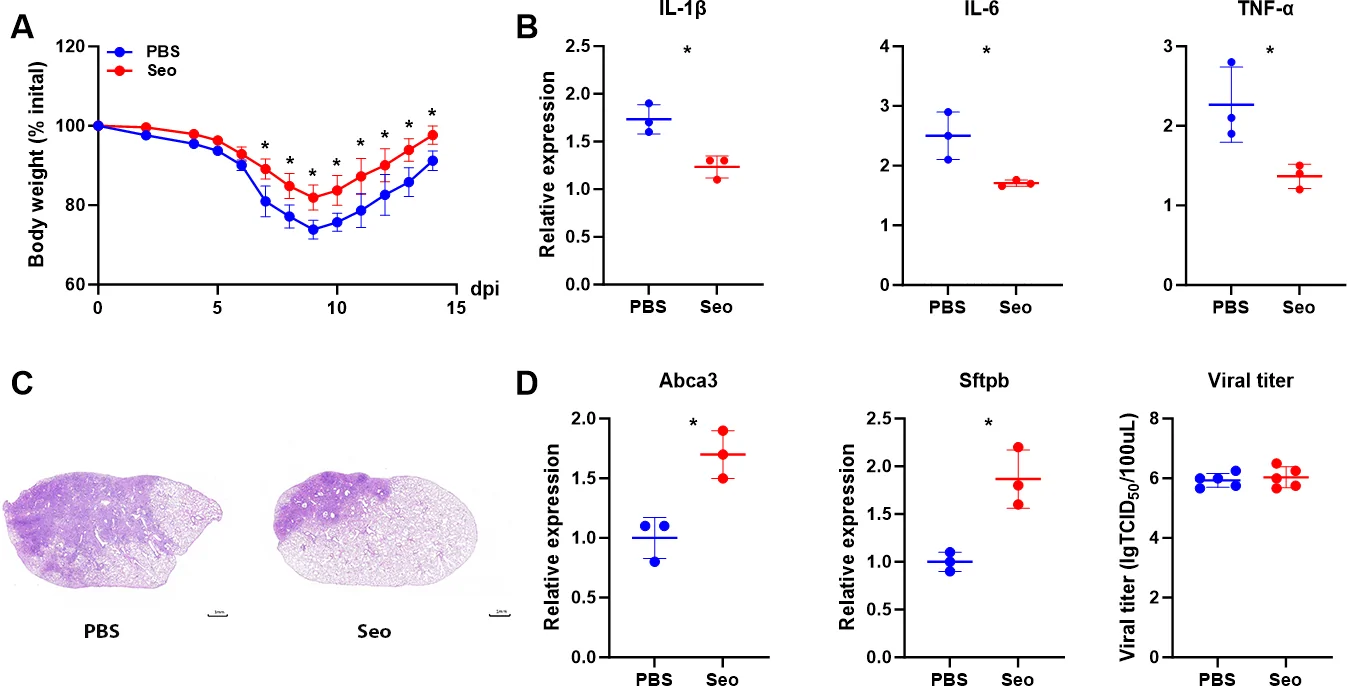
Seo concurrently reduces pulmonary inflammation and promotes tissue recovery in H9N2-infected mice. (**A**) Mice were infected with sublethal doses of H9N2 and treated with PBS or Seo (*n* = 10). Host mortality was monitored. (**B**) Lung inflammatory cytokine levels at 4 dpi were measured. (**C**) Viral titers were assessed at 4 dpi. (**D**) Gene expression levels of Abca3 and Sftpb in the lungs were analyzed at 14 dpi. Data are presented as means ± SEM. *P < 0.05.

To evaluate the *in vivo* anti-inflammatory effects of Seo, animal experiments were performed in a mouse model. Mice received intraperitoneal injections of either Seo or PBS prior to H9N2 AIV infection. Seo administration significantly alleviated disease symptoms post-infection (Fig. 7A; *P* < 0.05). Additionally, inflammatory gene expression in the lungs was markedly suppressed at 4 days post-infection (Fig. 7B; *P* < 0.05). Furthermore, the pathology result showed that Seo mitigates the inflammation and inflammatory cell infiltration caused by viral infection (Fig. 7C). Viral titers in the lungs of Seo-treated mice remained comparable to those in the control group (Fig. 7D). Following alveolar injury, alveolar type II (ATII) cells undergo proliferation and differentiation into alveolar type I (AT1) cells to facilitate lung epithelial repair (22). Notably, Seo-treated mice exhibited a significant increase in Abca3 and Sftpb expression, key ATII cell markers, at 14 dpi (Fig. 7C), indicating its role in promoting lung recovery (*P* < 0.05).

## DISCUSSION

H9N2 AIV is a prevalent low-pathogenic AIV that poses significant threats to both poultry and public health (2). While H9N2 infections in poultry often result in subclinical symptoms, co-infection with other pathogens can exacerbate disease severity, leading to economic losses in the poultry industry (3). More concerningly, H9N2 AIV is capable of crossing species barriers and has been detected in humans, where it can cause mild-to-severe respiratory infections (23). Given its potential to contribute to the genetic reassortment of more virulent influenza strains, the need for effective antiviral and anti-inflammatory strategies against H9N2 AIV is critical. In this study, we showed that HIF-1α contributes to the H9N2 AIV-induced inflammation. Furthermore, we identified that Seo is an effective HIF-1α inhibitor and can mitigate the viral-induced inflammation, thereby protecting the host from H9N2 infection.

HIF-1α is a master regulator of cellular responses to hypoxia and plays a crucial role in immune regulation, metabolic adaptation, and inflammation during viral infections (21). Numerous studies have indicated that viruses exploit the HIF-1α pathway to enhance viral replication and modulate the host immune response (9, 10). For example, viral infections can upregulate HIF-1α expression, which in turn promotes glycolysis and alters host cell metabolism to favor viral propagation (24). Additionally, HIF-1α has been implicated in driving excessive inflammatory responses, exacerbating lung injury during viral infections. Thus, targeting HIF-1α represents a promising therapeutic strategy for controlling both viral replication and inflammation. Here, we performed the loss-of-function and gain-of-function experiment to investigate the role of HIF-1α in H9N2 AIV pathogenesis. We found that HIF-1α has no effect on viral replication. However, HIF-1α contributes to the excessive inflammation caused by H9N2 AIV infection (Fig. 1 and 2).

Mitochondrial homeostasis plays a crucial role in regulating immune responses during viral infections. Influenza viruses, including H9N2 AIV, can disrupt mitochondrial function, leading to excessive ROS production, metabolic reprogramming, and inflammation (25). Mitochondrial damage triggers the release of mitochondrial DNA, which activates innate immune pathways, further amplifying pro-inflammatory cytokine production (26). Additionally, HIF-1α is stabilized under mitochondrial dysfunction, promoting inflammatory gene expression (27). Here, our study demonstrates that HIF-1α promotes H9N2-induced inflammation through disrupting mitochondrial homeostasis, which is evidenced by reduced respiration capacity and downregulated mitochondrial critical genes (Fig. 3 and 4).

Sotetsuflavone (Seo, a natural flavonoid compound, has been reported to exert anti-inflammatory effects through multiple signaling pathways (14, 15). We provide evidence that Seo effectively modulates the inflammatory response induced by H9N2 AIV infection, at least in part, through the inhibition of HIF-1α (Fig. 5 and 6). Network pharmacology analysis, molecular docking, and SPR analyses confirmed a strong binding affinity between Seo and HIF-1α, suggesting a direct interaction between them. Furthermore, our findings revealed that Seo treatment significantly reduced HIF-1α expression in infected cells, thereby limiting H9N2-induced inflammation. This is consistent with previous reports indicating that flavonoids can suppress HIF-1α activity and downstream inflammatory pathways (21).

In addition to its anti-inflammatory effects, Seo also appears to promote lung tissue recovery following viral infection. In our *in vivo* mouse model, Seo-treated mice exhibited reduced disease severity and suppressed inflammatory gene expression in the lungs. Furthermore, at 14 days post-infection, these mice showed increased expression of ATII cell markers Abca3 and Sftpb, suggesting enhanced lung epithelial repair. ATII cells play a crucial role in lung regeneration, as they proliferate and differentiate into AT1 cells, which are essential for alveolar gas exchange (22). The ability of Seo to promote ATII cell-associated lung repair underscores its potential as a therapeutic agent not only for mitigating inflammation but also for accelerating tissue recovery after influenza infection. Interestingly, despite Seo’s potent anti-inflammatory effects, our data indicate that Seo treatment did not significantly alter viral titers in the lungs of infected mice. This suggests that the primary mechanism of Seo’s protective effects is through modulating host immune responses rather than directly inhibiting viral replication.

Collectively, our findings provide strong evidence that Seo serves as an effective HIF-1α inhibitor, thereby alleviating H9N2-induced inflammation and promoting lung tissue recovery. Given the critical role of HIF-1α in viral pathogenesis and immune regulation, targeting this pathway could represent a promising therapeutic approach for treating influenza and other respiratory viral infections. Future studies should further explore the pharmacokinetics, safety, and long-term effects of Seo, as well as its potential synergistic effects with existing antiviral drugs. Understanding how Seo interacts with other key signaling pathways during influenza infection will be essential for optimizing its therapeutic application.

### Conclusion

**Fig 8:**
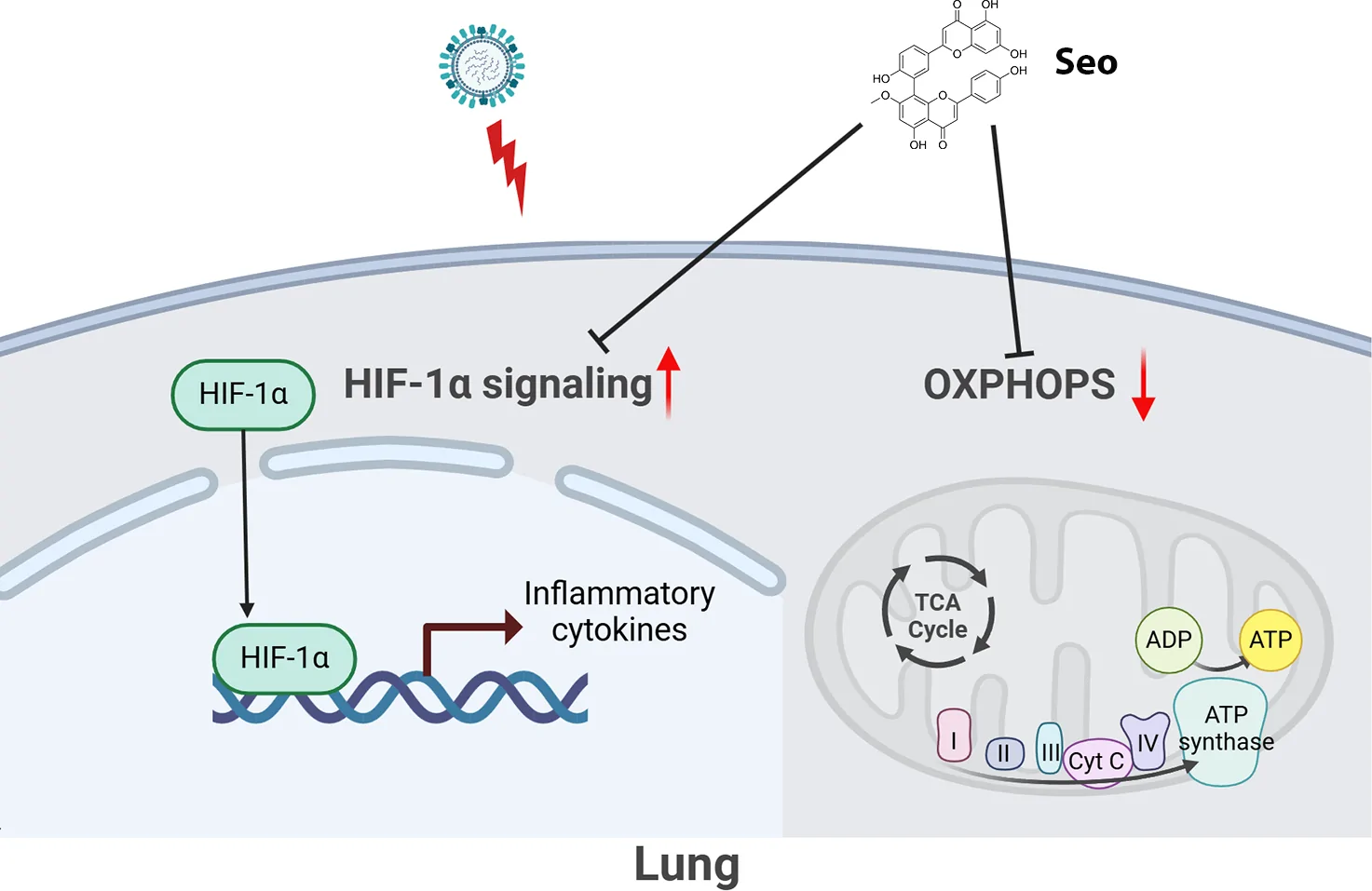
Work model of Seo. Seo mitigates H9N2-induced lung inflammation by suppressing the HIF-1α signaling pathway while preserving mitochondrial fitness.

In conclusion, our study highlights the potential of Seo as a novel therapeutic agent against H9N2 AIV infection. By inhibiting HIF-1α and suppressing excessive inflammation, Seo not only mitigates lung injury but also facilitates tissue repair (Fig. 8). These findings provide a basis for further investigation into the development of Seo-based treatments for influenza and other inflammatory respiratory diseases.

## Data Availability

Data will be made available on request.
